# Assessment of the Variation Associated with Repeated Measurement of Gastrointestinal Transit Times and Assessment of the Effect of Oral Ranitidine on Gastrointestinal Transit Times Using a Wireless Motility Capsule System in Dogs

**DOI:** 10.1155/2012/938417

**Published:** 2012-06-26

**Authors:** Jonathan A. Lidbury, Jan S. Suchodolski, Renata Ivanek, Jörg M. Steiner

**Affiliations:** ^1^Department of Small Animal Clinical Sciences, College of Veterinary Medicine & Biomedical Sciences, Texas A&M University, 4474 TAMU, College Station, TX 77843, USA; ^2^Department of Veterinary Integrative Biosciences, College of Veterinary Medicine & Biomedical Sciences, Texas A&M University, 4474 TAMU, College Station, TX 77843, USA

## Abstract

This study aimed to evaluate the variation associated with repeated measurement of gastrointestinal (GI) transit times and the effect of oral ranitidine on GI transit times in healthy dogs using a wireless motility capsule (WMC) system. Eight privately owned healthy adult dogs were enrolled, and one developed diarrhea and was removed from the study. For the first 3 repetitions, each dog was fed a standard meal followed by oral administration of a WMC. For the 4th repetition, each dog was given ranitidine hydrochloride (75 mg PO every 12 hours) prior to and during assessment of GI transit times. Mean between-subject coefficients of variation for gastric emptying time (GET), small and large bowel transit time (SLBTT), and total transit time (TTT) were 26.9%, 32.3%, and 19.6%, respectively. Mean within-subject coefficients of variation for GET, SLBTT, and TTT were 9.3%, 19.6%, and 15.9%, respectively. Median GET, SLBTT, and TTT without ranitidine were 719, 1,636, and 2,735 minutes, respectively. Median GET, SLBTT, and TTT with ranitidine were 757, 1,227, and 2,083 minutes, respectively. No significant differences in GI transit times were found between any of the 4 repetitions. Under these experimental conditions, no significant effects of oral ranitidine on GI transit times were observed.

## 1. Introduction

Although several techniques for assessing canine gastric emptying time (GET) have been reported, each of these has limitations [1]. Nuclear scintigraphy is currently considered to be the gold standard method for assessing GET in both humans [2] and dogs [1]. The radiopharmaceutical agents used for nuclear scintigraphy mix readily with the test meal, allowing this technique to provide a representative assessment of solid-phase gastric emptying. The need for specialized equipment, safety issues, and legislative implications of using a radioisotope means that this technique is rarely applied in clinical practice. Radiographic assessment of the exit of a barium based contrast agent from the stomach has also been used to assess canine gastric emptying [3, 4]. However, barium is not physically or chemically similar to the diet of a dog and separates from foodstuffs even when mixed with a meal. Consequently, this technique may not accurately reflect solid-phase gastric emptying. Barium-impregnated spheres may also be used to assess GET and may serve as a more representative marker than liquid barium [5, 6]. However, there are important differences in the rate and pattern of canine solid-phase gastric emptying of a radiolabeled meal as measured with barium-impregnated spheres and nuclear scintigraphy [5]. The utility of abdominal ultrasound for assessment of canine GET has been evaluated [7, 8]. This technique avoids the use of ionizing radiation, but it is time consuming and operator dependent. A ^13^C-octanoic acid breath test has been described for the assessment of canine GET [7, 9]. This technique does not involve ionizing radiation, and the breath samples collected could be analyzed at a distant reference laboratory. However, currently the effects of intestinal and hepatic disease on the performance of this test are not known.

Canine small intestinal, large intestinal, and whole gut transit times have been assessed by feeding dogs radiopaque markers and taking serial radiographs [10, 11]. However, techniques to quantitatively assess canine small intestinal and colonic motility are not routinely used in a clinical setting.

The SmartPill pH.p wireless motility capsule (WMC) system (SmartPill Corp., Buffalo, USA) measures pH, pressure, and temperature as it passes through the gastrointestinal tract. This data is transmitted wirelessly to a receiver allowing GET, small and large bowel transit time (SLBTT), and total GI transit time (TTT) to be calculated [12]. Gastric emptying of the WMC is indicated by a rapid increase in gastric pH. In humans, this system has also been validated for measurement of small intestinal transit time (STT) and colonic transit time (CTT) [13]. Transit of the WMC from the small intestine to the large intestine is indicated by a gradual decrease in pH and an alteration in the character of the pressure profile. Although these characteristic changes are often present in WMC system recordings from dogs, it has not been proven that the changes in pH and pressure waveform described above coincide with passage of the WMC from the small intestine to the large intestine in this species. This system has been shown to be a safe technique to assess GI transit times in dogs with body weights as low as 19.6 kg [14]. GETs measured by this technique correlate well with those (*r* = 0.76) measured by scintigraphy in healthy dogs [15]. Also, this system has the advantage of allowing GI transit times to be assessed in ambulatory patients in their home environments.

The variance of a set of data made up of serial results from a group of subjects is derived from the following components: preanalytical influences, analytical variation, and inherent biological variation. Biological variation is comprised of between-subject variation and within-subject variation [16]. When designing experiments such as assessing the effects of interventions, including therapies, it is important to consider all of these sources of variation. These sources of variation may limit the ability of tests of GI motility to distinguish between healthy and diseased patients or to detect the effect of interventions. The within-subject variation associated with measurement of GETs using this WMC system was recently shown to be equivalent to that measured by scintigraphy in healthy research dogs [17]. The variation associated with repeated measurement of intestinal transit times in privately owned dogs kept in the home environments has not previously been studied using this system.

Ranitidine is a histamine-2 receptor antagonist that suppresses histamine-stimulated gastric acid secretion [18]. Ranitidine also has an acetyl-cholinesterase inhibitor effect and consequently a prokinetic effect on GI smooth muscle [19, 20]. This drug has been shown to increase *in vivo* gastrointestinal motility dogs [21]. However, in humans, one study failed to find a significant effect of ranitidine on GET [22], while others found that ranitidine decreased GET [23–25]. A recent study failed to find a significant effect of ranitidine on GET in horses [26]. To the authors' knowledge, the effect of ranitidine on GET and other GI transit times in dogs has not previously been reported.

The first objective of this study was to assess the variation associated with repeated measurement of GI transit times in privately owned healthy adult dogs in their home environments using this WMC system. The second objective was to assess the effect of oral ranitidine on GI transit times in the same group of dogs using this WMC system.

## 2. Materials and Methods

### 2.1. Animals

Eight privately owned healthy adult dogs were enrolled in this study. The health of each dog was assessed using an owner questionnaire, a physical examination, a complete blood count, a serum biochemistry panel, and determination of serum pancreatic lipase concentration by Spec cPL ELISA Test (IDEXX Laboratories, Westbrook, USA), serum trypsin-like immunoreactivity concentration by RIA (Siemens Healthcare Diagnostics, Deerfield, USA), and serum folate and cobalamin concentrations using automated Immulite 2000 chemiluminescent assays (Siemens Healthcare Diagnostics, Deerfield, USA).

Inclusion criteria for the study were being 10 months of age or older and having a body weight greater than 20 kg. Dogs were excluded from the study if they had any ongoing disease or clinical signs of disease, if they had a history of past illness that was likely to be chronic, if they were currently receiving any medications other than routine prophylactic drugs, if they had any clinically important abnormalities on physical examination, or if they had any clinically important abnormalities noted after performing the blood tests above. 

The study was approved by the Texas A&M University Veterinary Medical Complex Clinical Research Review Committee, and informed owner consent was obtained for the enrollment of all the dogs.

### 2.2. Measurement of GI Transit Times

At least 2 weeks prior to entering the study, dogs were transitioned from their usual food onto Purina EN Gastroenteric Canine Formula dry dog food (Nestlé-Purina PetCare Co., St. Louis, USA), a commercially available easily digestible complete dry food. This diet and water were fed exclusively during the study with no treats allowed. Prior to testing, the amount of dry food fed to each dog was left at the owner's discretion.

**Figure 1:**
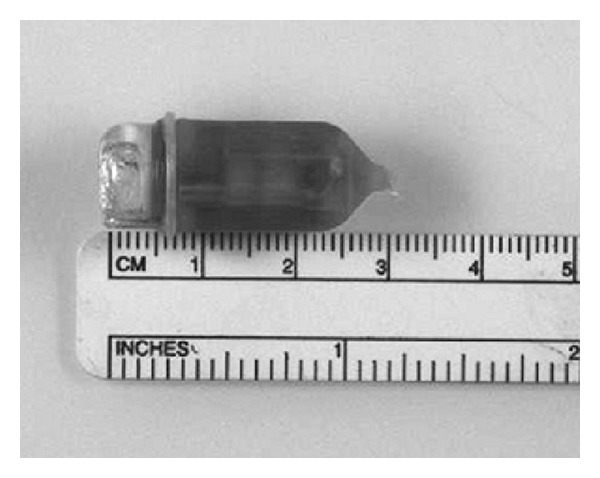
A SmartPill pH.p wireless motility capsule.

Each dog was tested on 4 occasions, approximately 1 week apart. Prior to each repetition, food but not water was withheld from the dogs for 12 hours. The following morning, the dogs were fitted with a vest containing the data receiver and were then fed a test meal of 185 g (approximately 705 kilocalories) of the dog food above. Immediately after the meal had been consumed, the WMC was administered orally to the dog. The WMCs weighed 4.0 g and are cylindrical in shape measuring 26 mm in length and 13 mm in diameter (Figure 1). The dog was then given the opportunity to drink up to 500 mL of water. The owner then took their dog home and was instructed to withhold water for 4 hours and food for 24 hours after administration of the WMC, not to let their dog swim, and not to vigorously exercise their dog. A diary of events such as meals, consumption of water, defecation, exercise, and passage of the WMC in the dog's feces was kept by the owner of the dog. 

To assess the variation associated with repeated measurements of GI transit times, the first 3 repetitions were performed without administering ranitidine. For the 4th repetition, each dog was administered 2 mg/kg (rounded up or down according to the available tablet sizes) of ranitidine hydrochloride (Zantac, Boehringer Ingelheim Corp., Ridgefield, USA) PO every 12 hours, starting 48 hours prior to administration of the WMC. The ranitidine was continued as above until the WMC was passed in the dog's feces. 

MotiliGI version 2.2 software (SmartPill Corp., Buffalo, USA) was used to analyze the data and calculate the following GI transit times: GET, STT, LTT, SLBTT, and TTT. The median pH in the stomach, small intestine, and colon was also calculated.

### 2.3. Statistical Analysis

Data were tested for normality by performing Kolmogorov-Smirnov tests while keeping in mind limitations of the test due to small sample size. Numerical data were described by the median followed by the range in parentheses. Between-subject and within-subject coefficients of variation (% CV) were calculated for GET, STT, LTT, SLBTT, and TTT for the first 3 time points. GI transit times and pHs for each anatomical region over the 4 time points were compared using repeated measures ANOVA or Friedman's tests if the data was parametric or nonparametric, respectively. Where significant differences between any time points were identified, post hoc testing was performed using Bonferroni's multiple comparisons tests or Dunn's tests if the data was parametric or nonparametric, respectively. Statistical significance was defined as *P* < 0.05. Prism version 5 (GraphPad Software Inc., San Diego, USA), a commercially available statistical software package, was used for all statistical calculations.

## 3. Results

Eight dogs were enrolled, 6 were female spayed, and 2 were male neutered. The median age of the dogs was 1.75 years (10 months to 11 years). Five of the dogs were of mixed breed, and there was one of each of the following breeds: Flat-coated Retriever, German Shepherd, and Golden Retriever. The median body weight of the dogs was 29.1 kg (22.1 to 41.0 kg). The median dose of ranitidine given was 2.6 mg/kg (1.8 to 3.4 mg/kg) PO every 12 hours.

**Figure 2:**
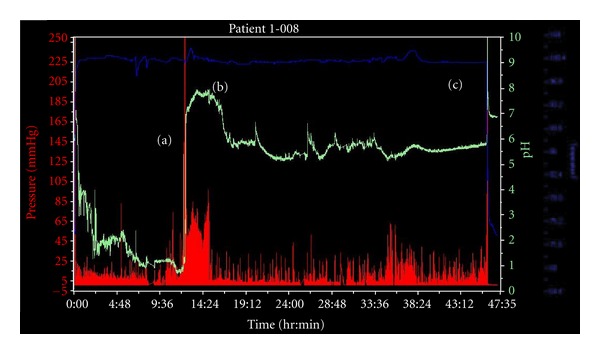
A representative WMC system recording from a healthy dog prior to treatment with ranitidine. The pH trace is shown in green, the pressure trace in red, and the temperature trace in blue. Gastric emptying is indicated by a rapid and sustained increase in pH (a) at 12 hours and 21 minutes, assumed passage of the WMC from the small intestine to the large intestine is represented by the onset of a gradual pH decrease and a change in the character of the pressure trace (b) at 15 hours and 14 minutes, and body exit of the WMC is marked by a sharp decrease in temperature (c) at 46 hours and 6 minutes.

A sharp rise in pH indicating exit of the WMC from the stomach was identified in each experiment. In 31/32 repetitions, it was possible to detect changes in pressure and pH consistent with passage of the WMC from the small intestine into the colon. Where the dog's owner observed passage of the WMC in the feces, it coincided with defecation of the WMC as calculated by observation of a rapid temperature change (Figure 2).

An 18-month-old female spayed mixed breed dog developed acute diarrhea during the third repetition, 13 hours after ingestion of the WMC. Oral ranitidine had not been given at the time, and the diarrhea resolved quickly without treatment. The intestinal transit times but not the GET of the WMC in this repetition were notably faster than for all other repetitions. For this repetition, it was not possible to locate the time at which this WMC passed from the small intestine into the large intestine. As this dog was not considered to be healthy at this point in time, it could not be used to assess variation in GI transit times for healthy dogs. It was not possible to remove just this repetition and analyze the data with repeated measures ANOVA or Friedman's test, so the GI transit times, rates of contraction, and intestinal pH for all 4 repetitions from this dog were removed from the analysis. The authors thought it was unlikely that gastric pH would be affected by acute diarrhea, so this data was included in the analysis. No other potential adverse events occurred during the study.

The between-subject % CV before treatment with ranitidine for GET, STT, LTT, SLBTT, and TTT was 26.9%, 29.2%, 35.4%, 32.3%, and 19.6%, respectively. The within-subject % CV before treatment with ranitidine for GET, STT, LTT, SLBTT, and TTT was 9.3%, 24.8%, 20.5%, 19.6%, and 15.9%, respectively (Table 1).

**Figure 3:**
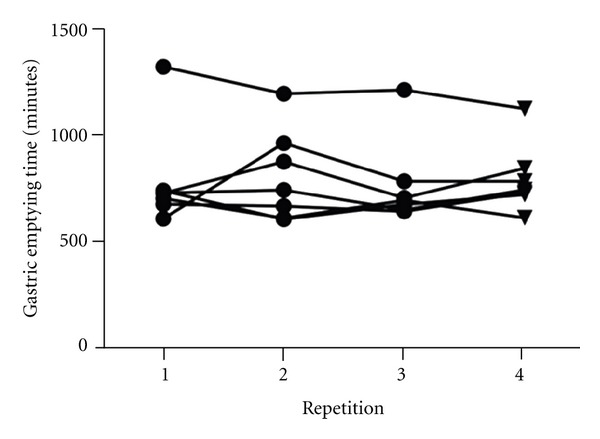
Gastric emptying times before and during treatment with ranitidine. The first 3 repetitions (circles) are before treatment with ranitidine, the 4th repetition (triangles) is during treatment with ranitidine, and there was no significant difference between any of the 4 repetitions (*P* = 0.6149).

The median (range) GET, STT, LTT, SLBTT, and TTT before treatment with ranitidine were 719 minutes (622–1,320 minutes), 183 minutes (92–290 minutes), 1,398 minutes (644–2,445 minutes), 1,636 minutes (746–2,588 minutes), and 2,735 minutes (1,898–3,296 minutes), respectively. The median GET, STT, LTT, SLBTT, and TTT during treatment with ranitidine were 757 minutes (628–1,128 minutes), 162 minutes (86–215 minutes), 1,140 minutes (342–2,481 minutes), 1,227 minutes (490–2,634 minutes), and 2,083 minutes (1,248–3,262 minutes), respectively. No significant differences in GET, STT, LTT, SLBTT, or TTT were found between any of the 4 time points (Table 1, Figure 3). The GET, SLBTT, and TTT for the dog that developed acute diarrhea before treatment with ranitidine were 979 minutes, 45 minutes, and 1025 minutes, respectively. It was not possible to calculate STT or LTT for this dog.

**Figure 4:**
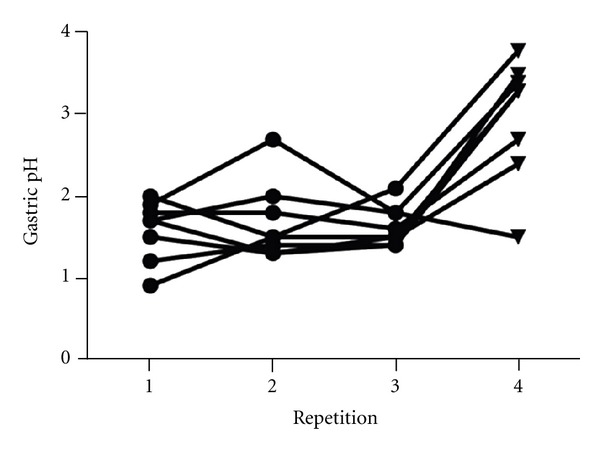
Gastric pH before and during treatment with ranitidine. The first 3 repetitions (circles) are before treatment with ranitidine, the 4th repetition (triangles) is during treatment with ranitidine, and gastric pH was lower for all 3 repetitions before treatment with ranitidine than the repetition during treatment with ranitidine (*P* < 0.001).

The median gastric pH, small intestinal pH, and large intestinal pH before treatment with ranitidine were 1.6 (0.9–2.7), 7.6 (7.3–8.2), and 5.7 (4.9–7.3), respectively. The median gastric pH, small intestinal pH, and colonic pH during treatment with ranitidine were 3.3 (1.5–3.8), 7.5 (7.3–8.1), and 6.0 (5.3–6.5), respectively. Gastric pH was significantly lower for all 3 repetitions prior to treatment with ranitidine than for the repetition during treatment with ranitidine (*P* < 0.001 for post hoc testing between each of the 3 time points prior to treatment and the time point during treatment; Figure 4). No significant differences between any of the 4 repetitions were found for small intestinal pH (*P* = 0.7553) or colonic pH (*P* = 0.6621).

## 4. Discussion

This WMC system is an easy-to-use, ambulatory, non-radioactive method for assessing GI transit times in dogs weighing more than 20 kg. Measurements of the GI transit times made using this system were subject to considerable between-subject and within-subject variation. Under these experimental conditions, no statistically significant effects of oral ranitidine on GI transit times were found. However, ranitidine caused a significant increase in gastric pH. No significant effects of ranitidine on small intestinal pH, or colonic pH, were found.

The measurements of GI transit times undertaken in this investigation were subject to considerable between-subject and within-subject variation. For example, the between-subject and within-subject % CV for TTT were 19.6% and 15.9%, respectively. The between-subject % CV for each transit time was higher than the within-subject % CV. It seems intuitive that there will be more variation associated with measurement of GI transit times among different dogs than with repeated measurements from the same dog. A previous study using 6 research dogs kept in a controlled environment found that the within-subject variation associated with repeated measurement of GETs using this WMC system were equivalent to that associated with measurement using nuclear scintigraphy [17]. The within-subject % CV for GET from the above study was 7.8%, which is similar to but slightly lower than the % CV of 9.3% from our study. This difference could be due to chance associated with the small number of dogs in both studies. However, as our study used privately owned dogs in their home environment rather than research dogs in a controlled environment, greater within-subject variation might be expected. Although owners were asked not to exercise their dogs vigorously during testing, the physical activity of the dogs in this study was not standardized. This could have led to increased variation in transit times as exercise has been shown to reduce GI motility [27]. Owners were asked to keep a diary of events such as exercising their dogs; however, insufficient detail was provided to allow investigation of the effect of exercise. As the within-subject mean % CV of dogs in our study was similar to that of the aforementioned group of dogs kept in a controlled environment [17], the effect on within-individual variability in GET was relatively minor. Furthermore, the fact that the variation associated with measurement of GET of privately owned dogs in their home environments was similar to that of dogs kept in a controlled environment is encouraging as it means the technique may be applicable to canine patients with a suspected GI motility disorder kept in their home environments. Stress has been shown to decrease GI motility in dogs [28], so if GI motility is to be assessed on a clinical rather than research basis, it would be preferable for the patient to be kept in their home rather than in the hospital environment during assessment. This WMC system is unique among techniques to assess GI transit times in that this is possible. The within-subject % CV associated with transit times involving the intestines (STT, LTT, SLBTT, and TTT) tended to be greater than those for GET. One factor that could contribute to the variation associated with measurement of LTT, SLBTT, and TTT is that defecation of the WMC is under voluntary control. The timing when these privately owned dogs were placed in a situation where they perceived it to be acceptable to defecate (as compared to dogs housed in a research facility) could have been an additional source of variation. The data from this study will be useful for calculating the sample sizes needed to achieve adequate statistical power in future studies using this system.

The median GETs calculated in our study and previous studies using this WMC system [14, 17] were longer than those previously reported using other techniques. For example, the median time for 95% a labelled meal to be emptied from the stomach of 27 dogs measured by nuclear scintigraphy was 148 minutes [29], whereas the median GET in our study was 719 minutes. This is not unexpected as the emptying of liquids from the stomach occurs before that of digestible solids, and once the digestible solids in the stomach have been triturated and emptied, large indigestible solids are emptied by interdigestive motor complexes [30]. The WMC used in this study is an indigestible solid and so exits the stomach after liquids and digestible solids. In humans, gastric emptying of WMCs has been shown to coincide with the onset of phase III migratingmyoelectrical complexes in the interdigestive period [31]. Nuclear scintigraphy measures the time it takes for a proportion (usually 50% or 90%) of a test meal comprised of digestible solids to be emptied from the stomach, and so GET measurements using this technique will be shorter than those made with the WMC system. However, in humans [12] and dogs (abstract by Andrews et al. [15]), GETs calculated using this system correlate with those calculated by nuclear scintigraphy. Consequently, although GETs measured using the WMC system are longer than those measured with scintigraphy, they may provide a similarly useful measurement. Indeed, in humans, this WMC system has been shown to have a diagnostic accuracy similar to that of nuclear scintigraphy for discriminating between patients with normal and delayed gastric emptying [12]. It is not currently known if the WMC system is as accurate as scintigraphy for diagnosing delayed gastric emptying in dogs, but this is worthy of future study.

An advantage of the WMC system over other techniques for assessment of GET is that it allows calculation of intestinal transit times. In humans, this WMC system has been validated for assessment of passage of the WMC from the small intestine to the large intestine and therefore for calculation of STT and LTT [13]. A gradual decrease in intestinal pH and alteration in pressure waveform indicating movement of the WMC from the small intestine into the large intestine was identified in 31/32 repetitions in this study, so it seems highly likely that the system can be used to measure STT and LTT in dogs. However, to the authors' knowledge, studies proving that these characteristic changes in pressure and pH coincide with movement of the WMC from the small intestine into the colon have not been performed in dogs. Therefore, readers should interpret data that is broken down into small intestine and colon in this paper cautiously.

To the authors' knowledge, the effect of ranitidine on GET in dogs has not previously been investigated. Under these experimental conditions, no statistically significant effects of oral ranitidine on GI transit times were found. Therefore, three possibilities must be considered. Firstly, it is possible that although ranitidine has previously been shown to increase canine GI motility *in vivo* [21], this drug does not have an effect on GI transit times because the increase in motility is insufficient to affect transit times. In humans, one study failed to find a significant effect of ranitidine on gastric emptying time [22], while others found that ranitidine decreased GET [23–25]. Secondly, this lack of a significant difference could be due to type II error, that is, ranitidine does make a difference, but the sample size in this study was too low to allow its detection. This study only used 8 dogs (the data from 1 of which was excluded from the analysis due to the development of acute diarrhea), and as previously discussed, the measurements of GI transit times were subject to considerable variation. However, there was no apparent trend towards being a treatment effect in the data. Finally, it may be that although ranitidine did not make a difference to GI transit times under these experimental conditions, under different experimental conditions, or in a different group of dogs, a treatment effect may have been observed. Healthy dogs were used in this study, and one could hypothesize that in order to determine whether or not a drug has a prokinetic effect, it will be more representative to test its effect on dogs with decreased GI motility. Another potentially important factor is the dose of ranitidine. The median dose of ranitidine given was 2.6 mg/kg PO every 12 hours with a range of 1.8 mg/kg to 3.4 mg/kg, which is close to the labeled dose of 2 mg/kg PO every 12 hours. It is possible that if a higher dose of ranitidine had been used, there would have been a detectable decrease in GI transit times. In conclusion, although no significant effects of ranitidine were observed in this study, the authors cannot reject the hypothesis that oral ranitidine decreases GI transit times in dogs. 

Ranitidine caused a modest but statistically significant increase in gastric pH. No significant effect of ranitidine on small intestinal or colonic pH was detected. This is not unexpected as ranitidine is a histamine-2 receptor antagonist and therefore decreases the production of acid by the oxyntic cells in the gastric glands. The efficacy of ranitidine for increasing gastric pH in dogs is controversial; one previous study found that ranitidine increases gastric pH in healthy dogs [32], while another found that ranitidine did not have a significantly different effect on gastric pH to saline when given IV [33]. It is important to state that our study was not designed to test the efficacy of ranitidine for the suppression of gastric acid production. The effect of ranitidine on gastric pH was not great enough or long lasting enough to prevent the WMC system from detecting movement of the capsule from the stomach into the duodenum.

One dog developed self-limiting acute diarrhea 13 hours after administration of the WMC. It seems unlikely that this was related to the test procedure. Interestingly, a 7-year-old female neutered, mixed breed dog, weighing 33.0 kg, that was part of a pilot study retained a WMC in her stomach for over 72 hours. Emesis was induced, by administering 0.02 mg/kg of apomorphine hydrochloride (Professional Compounding Centers of America, Houston, USA) compounded as 2 mg/mL solution IV, resulting in successful recovery of the WMC. To the author's knowledge, this is the first dog in which a gastric retention of a WMC has occurred. This dog was free from clinical signs, and no significant abnormalities were found on physical examination. Other studies using this WMC system have used dogs weighing as little as 19.6 kg. The WMC measures 26 mm in length and 13 mm in diameter, and so its administration to smaller patients or those with suspected mechanical obstruction of their GI tract is contraindicated. However, based on our experiences with this dog, WMC retention is still a risk in healthy dogs weighing more than 30 kg.

This study is subject to several limitations. Firstly, only 8 dogs were enrolled, one of which was removed from analysis due to the development of acute diarrhea. This may have led to insufficient power to detect an effect of ranitidine on GI transit times. Using a mean GET of 719 minutes, a standard deviation of 214 minutes, and a type I error rate of 5%, it is estimated that a sample size of 52 dogs in the control group and 52 dogs in the treatment group would be needed to have an 80% chance of detecting a 100-minute difference in GET between the groups. We only enrolled 8 dogs in this study due to cost. Additionally, at the time the study was designed, the data needed to perform sample size calculations for this technique had not been published. A period of acclimatization prior to the test procedure was not used to decrease patient stress. This could have increased the stress of the dogs, adversely affecting GI motility. However, the patients were sent home after being given the WMC and all tolerated wearing the receiver well, so the stress was minimized. Additionally, as ranitidine was given at the final test point, the dogs would be expected to have acclimatized by this time increasing the chances of detecting a treatment effect. Positive and negative controls were not used during the study. However, the authors do not consider it to be ethically acceptable to use a drug to cause GI stasis in privately owned dogs, and the dramatically increased rate of GI transit seen in the dog that developed diarrhea demonstrates that this method can detect the effects of increased GI motility. 

## 5. Conclusions

The WMC system used in this study is a useful method for assessing canine GI transit times in medium to large breed dogs. The system is easy to use and can be performed on ambulatory dogs in their home environments. However, even in healthy dogs weighing more than 30 kg, WMC retention is a risk. Considerable between-subject and within-subject variation in GI transit times occur in privately owned healthy dogs kept in their home environments. This study showed that the within-individual variation of GET in healthy privately owned adult dogs kept in their home environments was comparable to that previously reported in healthy research dogs kept in a controlled environment [17]. Although no significant effects of oral ranitidine on GI transit times were observed in this study, the authors cannot reject the hypothesis that oral ranitidine decreases GI transit times in dogs. The effect of higher doses of ranitidine and the effect of this and other prokinetic drugs on dogs with decreased GI motility are worthy of further study. Because of between-individual and within-individual variation in GI transit times, future studies need to have relatively large sample sizes.

## Figures and Tables

**Table 1:** Summary of GI transit times in healthy dogs measured using the WMC system before and during treatment with ranitidine. % CV = coefficient of variation. Between-subject and within-subject coefficients of variation were calculated based on measurements taken prior to treatment with ranitidine. *P* values are for testing the null hypothesis that transit time does not differ among the 4 repetitions (3 before treatment with ranitidine and the 4th during treatment with ranitidine). ^∗^: nonparametric data, transit times for the 4 repetitions were compared using a Friedman's test; ^∗∗^: parametric data, transit times for the 4 repetitions were compared using a repeated measures ANOVA.

Transit time	Between-subject % CV	Within-subject % CV	Median before ranitidine	Median with ranitidine	*P* value
			(range; minutes)	(range; minutes)	
Gastric emptying time	26.9%	9.3%	719 (622–1,320)	757 (628–1,128)	0.6149^∗^
Small intestinal	29.2%	24.8%	183 (92–290)	162 (86–215)	0.5007^∗∗^
Large intestinal	35.4%	20.5%	1,398 (644–2,445)	1,140 (342–2,481)	0.6172^∗∗^
Small and large intestinal	32.3%	19.6%	1,636 (746–2,588)	1,227 (490–2,634)	0.6215^∗∗^
Total gastrointestinal	19.6%	15.9%	2,735 (1,898–3,296)	2,083 (1,248–3,262)	0.2759^∗^

## References

[1] Wyse CA, McLellan J, Dickie AM, Sutton DG, Preston T, Yam PS (2003). A review of methods for assessment of the rate of gastric emptying in the dog and cat: 1898–2002. *Journal of Veterinary Internal Medicine*.

[2] Parkman HP, Harris AD, Krevsky B, Urbain JLC, Maurer AH, Fisher RS (1995). Gastroduodenal motility and dysmotility: an update on techniques available for evaluation. *The American Journal of Gastroenterology*.

[3] Miyabayashi T, Morgan JP (1984). Gastric emptying in the normal dog a contrast radiographic technique. *Veterinary Radiology*.

[4] Burns J, Fox SM (1986). The use of a barium meal to evalutae total gastric emptying time in the dog. *Veterinary Radiology*.

[5] Lester NV, Roberts GD, Newell SM, Graham JP, Hartless CS (1999). Assessment of barium impregnated polyethylene spheres (BIPS) as a measure of solid-phase gastric emptying in normal dogs-comparison to scintigraphy. *Veterinary Radiology and Ultrasound*.

[6] Nelson OL, Jergens AE, Miles KG, Christensen WF (2001). Gastric emptying as assessed by barium-impregnated polyethylene spheres in healthy dogs consuming a commercial kibble ration. *Journal of the American Animal Hospital Association*.

[7] McLellan J, Wyse CA, Dickie A, Preston T, Yam PS (2004). Comparison of the carbon 13-labeled octanoic acid breath test and ultrasonography for assessment of gastric emptying of a semisolid meal in dogs. *American Journal of Veterinary Research*.

[8] Chalmers AF, Kirton R, Wyse CA (2005). Ultrasonographic assessment of the rate of solid-phase gastric emptying in dogs. *Veterinary Record*.

[9] Wyse CA, Preston T, Love S, Morrison DJ, Cooper JM, Yam PS (2001). Use of the 13C-octanoic acid breath test for assessment of solid-phase gastric emptying in dogs. *American Journal of Veterinary Research*.

[10] Bruce SJ, Guilford WG, Hedderley DI, Mccauley M (1999). Development of reference intervals for the large intestinal transit of radiopaque markers in dogs. *Veterinary Radiology and Ultrasound*.

[11] Weber MP, Stambouli F, Martin LJ, Dumon HJ, Biourge VC, Nguyen PG (2002). Influence of age and body size on gastrointestinal transit time of radiopaque markers in healthy dogs. *American Journal of Veterinary Research*.

[12] Kuo B, McCallum RW, Koch KL (2008). Comparison of gastric emptying of a nondigestible capsule to a radio-labelled meal in healthy and gastroparetic subjects. *Alimentary Pharmacology & Therapeutics*.

[13] Rao SSC, Kuo B, McCallum RW (2009). Investigation of colonic and whole-gut transit with wireless motility capsule and radiopaque markers in constipation. *Clinical Gastroenterology and Hepatology*.

[14] Boillat CS, Gaschen FP, Hosgood GL (2010). Assessment of the relationship between body weight and gastrointestinal transit times measured by use of a wireless motility capsule system in dogs. *American Journal of Veterinary Research*.

[15] Andrews F, Denovo R, Reese R (2008). The evaluation of the wireless capsule (SmartPill) for measuring gastric emptying time and GI transit in normal dogs. *Journal of Veterinary Internal Medicine*.

[16] Fraser CG, Harris EK (1989). Generation and application of data on biological variation in clinical chemistry. *Critical Reviews in Clinical Laboratory Sciences*.

[17] Boillat CS, Gaschen FP, Gaschen L, Stout RW, Hosgood GL (2010). Variability associated with repeated measurements of gastrointestinal tract motility in dogs obtained by use of a wireless motility capsule system and scintigraphy. *American Journal of Veterinary Research*.

[18] Bohman T, Myren J, Larsen S (1980). Inhibition of the histamine-stimulated gastric secretion in healthy subjects by the H2-receptor antagonist ranitidine. *Scandinavian Journal of Gastroenterology*.

[19] Bertaccini G, Scarpignato C (1982). Histamine H2-antagonists modify gastric emptying in the rat. *British Journal of Pharmacology*.

[20] Bertaccini G, Poli E, Adami M, Coruzzi G (1983). Effect of some new H2-receptor antagonists on gastrointestinal motility. *Agents and Actions*.

[21] Fioramonti J, Soldani G, Honde C, Bueno L (1984). Effects of ranitidine and oxmetidine on gastrointestinal motility in conscious dog. *Agents and Actions*.

[22] Parikh R, Sweetland J, Forster ER, Bedding AW, Farr SJ, Smith JTL (1994). Ranitidine bismuth citrate and ranitidine do not affect gastric empyting of a radio-labelled liquid meal. *British Journal of Clinical Pharmacology*.

[23] Scarpignato C, Bertaccini G (1982). Different effects of cimetidine and ranitidine on gastric emptying in rats and man. *Agents and Actions*.

[24] Houghton LA, Read NW (1987). A comparative study of the effect of cimetidine and ranitidine on the rate of gastric emptying of liquid and solid test meals in man. *Alimentary Pharmacology & Therapeutics*.

[25] Amir I, Anwar N, Baraona E, Lieber CS (1996). Ranitidine increases the bioavailability of imbibed alcohol by accelerating gastric emptying. *Life Sciences*.

[26] Maher O, Nieto JE, Stanley SD, Dore E, Snyder JR (2008). Evaluation of the effect of ranitidine on gastroduodenal contractile activity and gastric emptying in horses. *American Journal of Veterinary Research*.

[27] Kondo T, Naruse S, Hayakawa T, Shibata T (1994). Effect of exercise on gastroduodenal functions in untrained dogs. *International Journal of Sports Medicine*.

[28] Mistiaen W, Blockx P, van Hee R, Bortier H, Harrisson F (2002). The effect of stress on gastric emptying rate measured with a radionuclide tracer. *Hepato-Gastroenterology*.

[29] van den Brom WE, Happ RP (1986). Gastric emptying of a radionuclide-labeled test meal in healthy dogs: a new mathematical analysis and reference values. *American Journal of Veterinary Research*.

[30] Hinder RA, Kelly KA (1977). Canine gastric emptying of solids and liquids. *The American Journal of Physiology*.

[31] Cassilly D, Kantor S, Knight LC (2008). Gastric emptying of a non-digestible solid: assessment with simultaneous SmartPill pH and pressure capsule, antroduodenal manometry, gastric emptying scintigraphy. *Neurogastroenterology and Motility*.

[32] Polentarutti B, Albery T, Dressman J, Abrahamsson B (2010). Modification of gastric pH in the fasted dog. *Journal of Pharmacy and Pharmacology*.

[33] Bersenas AME, Mathews KA, Allen DG, Conlon PD (2005). Effects of ranitidine, famotidine, pantoprazole, and omeprazole on intragastric pH in dogs. *American Journal of Veterinary Research*.

